# Phylogeography and Population Genetics Analyses Reveal Evolutionary History of the Desert Resource Plant *Lycium ruthenicum* (Solanaceae)

**DOI:** 10.3389/fpls.2022.915526

**Published:** 2022-06-30

**Authors:** Gulbar Yisilam, Chen-Xi Wang, Mao-Qin Xia, Hans Peter Comes, Pan Li, Jin Li, Xin-Min Tian

**Affiliations:** ^1^Xinjiang Key Laboratory of Biological Resources and Genetic Engineering, College of Life Science and Technology, Xinjiang University, Urumqi, China; ^2^Xinjiang Key Laboratory of Special Species Conservation and Regulatory Biology, Key Laboratory of Plant Stress Biology in Arid Land, College of Life Science, Xinjiang Normal University, Urumqi, China; ^3^Laboratory of Systematic & Evolutionary Botany and Biodiversity, College of Life Sciences, Zhejiang University, Hangzhou, China; ^4^Department of Environment and Biodiversity, University of Salzburg, Salzburg, Austria

**Keywords:** phylogeography, desert plants, *Lycium ruthenicum*, genetic structure, quaternary

## Abstract

Climactic oscillations during the Quaternary played a significant role in the formation of genetic diversity and historical demography of numerous plant species in northwestern China. In this study, we used 11 simple sequence repeats derived from expressed sequence tag (EST-SSR), two chloroplast DNA (cpDNA) fragments, and ecological niche modeling (ENM) to investigate the population structure and the phylogeographic history of *Lycium ruthenicum*, a plant species adapted to the climate in northwestern China. We identified 20 chloroplast haplotypes of which two were dominant and widely distributed in almost all populations. The species has high haplotype diversity and low nucleotide diversity based on the cpDNA data. The EST-SSR results showed a high percentage of total genetic variation within populations. Both the cpDNA and EST-SSR results indicated no significant differentiation among populations. By combining the evidence from ENM and demographic analysis, we confirmed that both the last interglacial (LIG) and late-glacial maximum (LGM) climatic fluctuations, aridification might have substantially narrowed the distribution range of this desert species, the southern parts of the Junggar Basin, the Tarim Basin, and the eastern Pamir Plateau were the potential glacial refugia for *L. ruthenicum* during the late middle Pleistocene to late Pleistocene Period. During the early Holocene, the warm, and humid climate promoted its demographic expansion in northwestern China. This work may provide new insights into the mechanism of formation of plant diversity in this arid region.

## Introduction

Global climate has fluctuated greatly during the Quaternary Period, leading to the alternation of glacial and interglacial periods (Hewitt, 2000, 2004). During glacial periods, cold, arid climates caused large-scale species extinction or migration to refugial locations. During interglacial periods, however, warm, moist climates promoted the rapid range expansion and recolonization of species (Hewitt, 1996; Muellner-Riehl, 2019). An inescapable consequence of these climatic oscillations for most species is great changes in their geographical distribution (Hewitt, 2000). Several phylogeographical studies have indicated that Quaternary climatic oscillations resulted in the expansions and intraspecific divergences in most plant species in China (Qiu et al., 2011; Liu et al., 2012a) such as *Picea crassifolia* (Meng et al., 2007), *Allium przewalskianum* (Wu et al., 2010), *Pteroceltis tatarinowii* (Li et al., 2012).

In the past, most plant phylogeographical studies in China have focused on the Hengduan Mountains and adjacent regions, in the southeast of the Qinghai-Tibetan Plateau (QTP; Meng et al., 2007; Cun and Wang, 2010). This area is referred to as the core of the greatest concentrations of biodiversity in the world (Myers et al., 2000). In recent years, several studies paid attention to the phylogeographic patterns and demographical history of desert plants near the northern edge of the QTP in the arid northwestern area of China. However, few studies have been undertaken concerning the roles of the Quaternary geology and the climatic oscillations in driving the population evolutionary history of plant species in arid northwestern China (Meng et al., 2015).

Ice sheets did not cover northwestern China, and the evolution of desert plant species, such as *Caragana* (Zhang and Fritsch, 2010), *Nitraria sphaerocarpa* (Su and Zhang, 2013), *Ribes meyeri* (Xie and Zhang, 2013) and *Aconitum nemorum* (Jiang et al., 2014), have been impacted by significant climatic oscillations (Li, 1998; Meng et al., 2015). Previous phylogeographical studies showed that the uplift of the QTP since the Cenozoic is one of the most important geological events, which has changed the global climate and intensified the aridification of northwestern China (Liu et al., 2014a, 2019). During the Pleistocene (*ca*. after 2.6 million years ago: Mya), continuous uplift of the QTP, the Tianshan, and the Himalayas coupled with weakening of the southwest monsoon and intensification of the plateau winter monsoon caused extreme drought and low temperatures (Willis and Niklas, 2004; Meng and Zhang, 2013; Liu et al., 2014a). Intensification of the East Asian winter monsoon intensified aridification and thus profoundly changed the hydrology and climate of this area (An et al., 2001; Guo et al., 2002; Miao et al., 2012). Hence, it notably led to accelerating the range expansion of the deserts such as the Gurbantunggut Desert of the Junggar Basin, Taklamakan Desert of the Tarim Basin, and Badain Jaran–Tengger desert to the north of the Hexi Corridor (Meng and Zhang, 2013; Yin et al., 2015). Climatic fluctuations, aridification, and desert expansion that developed in the Quaternary appear to have played a significant role in determining the phylogeographic patterns and demographical history of numerous desert plants species in these areas (Chen et al., 2008a; Su et al., 2016; Wang et al., 2016a; Zhao et al., 2019; Zhang et al., 2020). For example, the divergence of *Larix sibirica* at approximately 1.6 mya in the Altai Mountains and the eastern Tianshan correlates to enhanced aridity in the Asian interior and the desert expansion in the Junggar Basin during the early Pleistocene (Zhang et al., 2014). Additionally, during the glacial periods, many species in these arid regions have retreated to refugial locations such as the southwestern margin of Junggar Basin, the Tarim Basin, and the Tianshan Mountains. These basins and mountains provided suitable living environments for desert plants such as *Libanotis buchtormensis* (Wang et al., 2016a), and *Atraphaxis frutescens* (Xu and Zhang, 2015).

*Lycium ruthenicum* Murr. (Solanaceae) is diploid, monoecious, and cross-pollinated and its root propagates *via* clones (Chen et al., 2008b; Kuang and Lu, 2011; Dai et al., 2013). It is widely distributed in the saline deserts, sands, and roadsides of northwestern China (Zheng et al., 2011; Zhao et al., 2021). *Lycium ruthenicum* produces relatively small, fleshy blackish fruits and its seeds are dispersed by wind, birds and rodents (Hitchcock, 1932; Alitong et al., 2013; Levin and Miller, 2021). *Lycium ruthenicum* berries have anti-cancer, anti-inflammatory, antioxidant, radioprotective, anti-aging, anti-fatigue, cardioprotective, neuroprotective, hepatoprotective, and hypolipidemic efficacy (Hu et al., 2014; Liu et al., 2014b; Duan et al., 2015; Tian et al., 2021; Wang et al., 2021). It has been overexploited and its wild resources are being destroyed. For these reasons, *L. ruthenicum* is now on the list of National Key Protected Wild Plants in China.^1^ This salt- and drought-tolerant shrub has high medicinal value and confers environmental protection in the arid western region of China (Lei et al., 2021). Thus, its distribution and biological characteristics make it an ideal candidate for phylogeographical studies. Studies on the genetics and conservation of *L. ruthenicum* have already been conducted (Liu et al., 2012b; Chen et al., 2014, 2017). Nevertheless, these prior works only used relatively less representative sequence-related amplified polymorphism (SRAP) markers, cpDNA and small sample sizes that did not suffice to elucidate the phylogeography of *L. ruthenicum* across its entire distribution range.

The aim of this study was to clarify the phylogeography of the taxa by determining how the distribution of *L. ruthenicum* changed during the Quaternary period. To this end, we used ecological niche modeling (ENM) and phylogeographical analyses based on the simple sequence repeats (SSRs) derived from expressed sequence tag (EST-SSR) and chloroplast DNA (cpDNA) fragments (*rps16*–*trnK* and *trnH*–*psbA*). We investigated the genetic diversity patterns of *L. ruthenicum* in northwestern China, established its historical population demography in response to climatic oscillations, and provided critical genetic information required to conserve it. The present work will serve as an empirical model for the study of desert plant phylogeography in northwestern China.

## Materials and Methods

### Population Sampling

Fresh, healthy leaves of 563 individuals, from 50 populations of *L. ruthenicum* in Xinjiang, Qinghai, Gansu, and Inner Mongolia, China, were collected and dried with silica gel (Table 1; Supplementary Table S1). Voucher specimens were deposited at the Herbarium of Xinjiang Normal University (XJNU). As this species can propagate by clonal growth, individuals >50 m apart were collected to avoid inadvertently harvesting clones (Wang et al., 2019).

**Table 1:** Details of sample localities for the 50 *Lycium ruthenicum* populations studied.

Population code	General collection site	Latitude (N)	Longitude (E)	*N*S (SSR/cpDNA)	EST-SSR					cpDNA sequences			
					*N*_A_	*A*_R_	*H_O_*	*H*_E_	*F*_IS_	*N*_C_	*H*d	*Pi* (×10^−3^)	Hap
**Eastern group**													
AQ	Doulan, Haixi, Qinghai	37°00′	96°51′	7/2	25	1.264	0.286	0.267	0.008^*^	2	1.000	0.900	H2 (1),H4 (1)
GD	Dunhuang, Gansu	40°13′	95°11′	12/5	24	1.290	0.318	0.283	−0.032	2	0.400	0.720	H3 (1),H4 (4)
GF	Wuwei, Gansu	39°10′	103°03′	14/5	31	1.425	0.403	0.355	0.265	3	0.700	1.080	H4 (3),H7 (1),H8 (1)
GH	Wuwei, Gansu	39°05′	103°12′	10/4	28	1.367	0.464	0.345	0.097	3	0.833	1.050	H2 (1),H4 (1), H9 (2)
GL	Jiuquan, Gansu	40°38′	96°04′	11/5	27	1.370	0.421	0.377	−0.046	3	0.800	1.260	H3 (1),H4 (2),H8 (2)
GM	Wuwei, Gansu	39°11′	103°03′	0/2	–	–	–	–	–	1	0.000	0.000	H3 (2)
GN	Jiuquan, Gansu	40°43′	96°07′	10/3	23	1.323	0.318	0.352	−0.226	1	0.000	0.000	H4 (3)
GO	Zhangye, Gansu	39°26′	100°59′	14/5	35	1.331	0.403	0.366	−0.330	1	0.000	0.000	H4 (5)
GQ	Jiayuguan, Gansu	40°25′	98°18′	11/5	15	1.300	0.298	0.411	−0.416	3	0.700	1.080	H4 (3),H9 (1),H10 (1)
GS	Wuwei, Gansu	39°02′	103°03′	7/4	19	1.451	0.468	0.346	0.024	3	0.833	0.900	H2 (1),H4 (2),H8 (1)
GT	Wuwei, Gansu	39°03′	103°02′	0/3	–	–	–	–	–	2	0.667	1.200	H9 (2),H11 (1)
GY	Zhangye, Gansu	39°38′	99°44′	13/5	68	1.397	0.406	0.385	0.149	3	0.800	1.620	H3 (2),H4 (1),H8 (2)
JC	Jiuquan, Gansu	40°08′	99°13	13/5	24	1.375	0.462	0.316	0.028	2	0.600	0.540	H4 (2),H8 (3)
JQ	Jiuquan, Gansu	39°37′	99°05′	13/5	28	1.322	0.434	0.335	−0.440	2	0.400	0.360	H4 (4),H10 (1)
JT	Jiuquan, Gansu	39°36′	99°03′	13/5	32	1.340	0.392	0.330	−0.398	3	0.800	1.620	H4 (1),H9 (2),H10 (2)
JX	Jiuquan, Gansu	39°34′	99°05′	11/5	34	1.375	0.438	0.380	−0.011	2	0.600	1.080	H3 (2),H4 (3)
JZ	Jiuquan, Gansu	40°08′	99°16′	14/5	36	1.373	0.409	0.438	0.127	3	0.700	1.260	H3 (1),H4 (3),H9 (1)
KA	Inner Mongolia	41°15′	103°54′	12/11	26	1.390	0.379	0.455	−0.185	2	0.182	0.160	H4 (10),H8 (1)
QC	Haixi, Qinghai	38°06′	95°18′	10/3	21	1.369	0.445	0.351	0.078	1	0.000	0.000	H4 (3)
QF	Greermu, Qinghai	36°37′	95°14′	0/4	–	–	–	–	–	3	0.833	2.250	H2 (2),H4 (1),H12 (1)
QG	Nuomuhong, Qinghai	36°39′	96°46′	9/0	22	1.291	0.414	0.269	0.048	–	–	–	
QL	Nuomuhong, Qinghai	36°45′	96°47′	0/1	–	–	–	–	–	1	0.000	0.000	H12 (1)
QM	Haixi, Qinghai	38°05′	94°53′	9/3	29	1.318	0.313	0.384	0.134	1	0.000	0.000	H4 (3)
QX	Haixi, Qinghai	37°25′	95°36′	6/5	31	1.423	0.333	0.278	0.265	3	0.700	2.340	H2 (3),H16 (1),H17 (1)
QY	Greermu, Qinghai	36°46′	95°20′	16/4	33	1.334	0.295	0.382	0.159	2	0.667	0.600	H2 (2),H4 (2)
XB	Jiuquan, Gansu	40°25′	99°09′	8/5	31	1.311	0.295	0.345	−0.077	2	0.400	0.360	H2 (1),H4 (4)
ZB	Jiuquan, Gansu	40°26′	99°07′	15/5	19	1.406	0.315	0.387	−0.014	3	0.800	1.440	H3 (2),H4 (1),H9 (2)
ZH	Zhangye, Gansu	39°38′	99°47′	0/2	–	–	–	–	–	1	0.000	0.000	H4 (2)
ZY	Zhangye, Gansu	39°36′	99°45′	0/4	–	–	–	–	–	2	0.667	1.200	H3 (2),H4 (2)
Regional level average					28.739	1.354	0.379	0.354	−0.034		0.522	0.853	
**Southern group**													
AK	Akto, Xinjiang	39°00′	76°26′	17/4	29	1.272	0.209	0.255	0.303	1	0.000	0.000	H1 (4)
AT	Atushi, Xinjiang	40°15′	75°58′	13/5	41	1.427	0.483	0.283	0.197	3	0.700	1.260	H1 (1),H3 (3),H5 (1)
HJ	Hejing, Xinjiang	42°54′	86°24′	0/1	–	–	–	–	–	1	0.000	0.000	H4 (1)
JS	Payzawat, Xinjiang	39°32′	77°16′	15/4	27	1.288	0.515	0.389	−0.530	4	1.000	2.700	H2 (1),H3 (1),H4 (1),H12 (1)
MG	Makit, Xinjiang	39°21′	78°02′	7/3	33	1.305	0.390	0.327	−0.455	2	0.667	2.400	H2 (1),H12 (2)
MY	Karakax, Xinjiang	37°20′	80°23′	19/5	25	1.373	0.407	0.396	0.371	4	0.900	2.160	H2 (2),H4 (1),H12 (1),H15 (1)
SC	Yarkant, Xinjiang	38°41′	77°27′	15/5	16	1.432	0.467	0.372	0.366	2	0.400	1.080	H2 (4),H18 (1)
TM	Tiemenguan Xinjiang	42°12′	86°17′	15/6	23	1.371	0.503	0.315	−0.366	1	0.000	0.000	H4 (6)
TS	Tashkurgan, Xinjiang	38°22′	75°20′	11/5	25	1.275	0.314	0.318	−0.523	4	0.900	1.620	H2 (2),H3 (1),H13 (1),H18 (1)
YJ	Yengisar, Xinjiang	39°01′	76°42′	15/5	42	1.345	0.345	0.356	−0.334	4	0.900	3.060	H2 (1),H3 (1),H12 (1),H19 (2)
YP	Yopurga, Xinjiang	39°21′	77°17′	21/6	32	1.401	0.398	0.430	0.100	2	0.600	2.220	H2 (4),H12 (1), H20 (1)
Regional level average					29.3	1.349	0.391	0.339	−0.087		0.607	1.650	
**Northern group**													
AL	Alashan Xinjiang	85°52′	40°37′	20/4	44	1.547	0.264	0.530	0.557	2	0.500	0.450	H2 (3),H3 (1)
BH	Habahe, Xinjiang	48°06′	86°43′	0/2	–	–	–	–	–	2	1.000	0.880	H4 (1),H6 (1)
BL	Bole, Xinjiang	45°10′	83°04′	15/5	27	1.353	0.521	0.319	−0.540	1	0.000	0.000	H4 (3)
CJ	Changji, Xinjiang	44°28′	87°25′	21/5	35	1.427	0.502	0.397	0.336	2	0.600	0.540	H6 (2),H7 (3)
HT	Hutubi, Xinjiang	44°29′	86°22′	20/3	31	1.381	0.468	0.382	0.250	2	0.667	0.600	H6 (1),H7 (2)
MN	Manas, Xinjiang	40°43′	86°18′	21/4	36	1.361	0.316	0.422	0.045	3	0.833	2.550	H4 (1),H13 (2),H14 (1)
QJ	QiJiaoJing, Xinjiang	43°48′	91°48′	20/5	15	1.306	0.309	0.421	−0.560	2	0.400	0.360	H4 (4),H8 (1)
SH	Shihezi, Xinjiang	44°48′	86°07′	0/1	–	–	–	–	–	1	0.000	0.000	H4 (1)
SW	Shawan, Xinjiang	45°00′	86°10′	17/5	17	1.289	0.380	0.337	−0.553	4	0.833	1.050	H2 (2),H4 (1),H10 (1),H13 (1)
TK	Toksun, Xinjiang	43°20′	89°07′	0/3	–	–	–	–	–	1	0.000	0.000	H3 (3)
Regional level average					29.286	1.381	0.394	0.401	−0.066		0.537	0.714	
Average					28.975	1.357	0.384	0.359	−0.053	19	0.776	1.52	

N_S_, number of individuals sampled; N_A_, number of EST-SSR alleles sampled; A_R_, allelic richness; H_O_, observed heterozygosity; H_E_, expected heterozygosity; F_IS_, inbreeding coefficient; N_C_, number of cpDNA haplotypes; H_d_, haplotype diversity; Pi, nucleotide diversity; and Hap, haplotypes (individual number) for each population. Asterisks denote significant deviation from Hardy–Weinberg equilibrium tested with 1,000 randomizations (*p* < 0.01).

### DNA Extraction, PCR Amplification, and Sequencing

Total DNA was extracted with DNA PLANTzol Reagent (Invitrogen, Carlsbad, CA, United States) according to the manufacturer’s protocol. DNA product quality and concentration were evaluated by 1% agarose gel electrophoresis. The DNA was stored at −20°C until use. For the EST-SSR, 11 polymorphic EST-SSR markers were screened and their corresponding primer pairs were developed for 540 individuals (Table 1; Supplementary Table S2). The primer sequences and amplification conditions for each primer set were previously described (Chen et al., 2017). For all loci, a forward primer was synthesized with the M13 sequence (5’-TGTAAAACGACGGCCAGT-3′) at the 5′ end and universal M13 primers (5’-TGTAAAACGACGGCCAGT-3′) were labeled with a fluorophore (FAM, TAMRA, HEX, or ROX) during PCR amplification (Tsingke Biotech Co., Beijing, China). Whole primers were used for two-step PCR amplification on 540 individuals from 40 populations (Schuelke, 2000). In the first step, the PCR was performed in a 15 μl reaction volume consisting of 1 μl template DNA, 7.5 μl of 2 × PCR Master Mix (Tsingke Biotech Co., Beijing, China), 5.5 μl deionized water, and 0.5 μl of each forward and reverse primer synthesized with M13. The PCR conditions involved an initial denaturation at 95°C for 5 min followed by 35 cycles at 95°C for 30 s, a locus-specific annealing temperature (Supplementary Table S2) for 30 s, 72°C for 30 s, and a final extension at 72°C for 10 min. In the second PCR step, the reaction volume of 30 μl consisted of ~3 μl product from the first PCR step, 15 μl of 2 × PCR Master Mix, 10 μl deionized water, 1 μl forward primer, and 1 μl fluorophore-labeled (FAM, ROX, HEX, or TAMRA) 18-bp M13 primer. The PCR conditions involved an initial denaturation at 94°C for 2 min followed by 35 cycles at 94°C for 60 s, 59°C for 45 s, 72°C for 1 min, and a final extension at 72°C for 10 min. The final fragment lengths of the PCR products were analyzed on an ABI 3730xl DNA Analyzer (Applied Biosystems, Foster City, CA, United States) using GeneScan 500 LIZ (Applied Biosystems) as an internal reference. Alleles and genotypes were identified with GeneMarker v. 2.2.0 (SoftGenetics, State College, PA, United States).

For the cpDNA, the primer pairs *rps16*–*trnK*, *trnH*–*psbA*, *matK*, and *rp132* from representative samples of *L. ruthenicum* were used in preliminary screening to detect variations in the cpDNA fragments (Shaw et al., 2005). Only the chloroplast intergenic fragments *rps16*–*trnK* and *trnH*–*psbA* had comparatively high levels of variation. Hence, they were examined in the subsequent analysis. The *trnH*–*psbA* and *rps16*–*trnK* for 206 samples from 49 populations were amplified and sequenced according to the methods of Shaw et al. (2005, 2007). The PCR conditions involved an initial denaturation at 94°C for 2 min followed by 38 cycles at 94°C for 1 min, a locus-specific annealing temperature (Supplementary Table S3) for 45 s, 72°C for 1 min, and a final extension at 72°C for 10 min. PCR products were sequenced by the Tsingke Biological Technology Co. (Beijing).

### Microsatellite Diversity and Population Structure Analyses

The number of alleles, allelic richness (*A*_R_), observed heterozygosity (*H*_O_), expected heterozygosity (*H*_E_), and inbreeding coefficient (*F*_IS_) for each population were calculated by FSTAT v. 2.9.3.2 (Goudet, 2001).^2^ The significance of the Hardy–Weinberg equilibrium was tested by 1,000 randomizations and the resulting values of *p* were subjected to Bonferroni correction in FSTAT v. 2.9.3.2 (Goudet, 2001). The population structure analysis was performed in STRUCTURE v. 2.3.3.^3^ The model supporting population admixture and correlated allele frequency was applied (Gilbert et al., 2012). The *K* values were in the range of 1–10 and there were 10 permutations per *K* value. STRUCTURE was run with 1,000,000 burn-in generations followed by 1,000,000 MCMC iterations. The *∆K*-value was obtained by the method of Evanno et al. (2005) to estimate the optimal genetic cluster (*K*) value. To this end, the online program STRUCTURE HARVESTER was used.^4^

### Haplotype Genealogy Analyses

All cpDNA (*rps16*–*trnK* and *trnH*–*psbA*) sequences were assembled and manually checked in Geneious v. 11.0.2.^5^ The sequences were aligned with MAFFT v. 7.450 (Katoh and Standley, 2013).^6^ *Lycium dasystemum* was sequenced and served as the outgroup in the chloroplast genealogy analysis (GenBank accession numbers, *rps16*–*trnK*: ON055439, *trnH*–*psbA*: ON055440). Haplotypes were determined using DnaSP v. 5.1 (Librado and Rozas, 2009).^7^ Then, the genealogical relationships among the cpDNA haplotypes were inferred by a median-joining network (Bandelt et al., 1999) with Network v. 10.2.^8^ In the analysis, each indel and inversion were treated as a single mutation event. The geographical distribution of the cpDNA haplotypes was mapped with ArcMap v. 10.6 (ESRI, Redlands, CA, United States). All *L. ruthenicum* haplotype sequences were deposited in GenBank under accession numbers ON390854–ON390893.

### Genetic Diversity and Population Genetic Structure Analyses

The nucleotide diversity based on the cpDNA sequences was estimated with DnaSP v. 5.1. Permut v.1.2.1 was used to compare *G*_ST_ and *N*_ST_ for the population phylogeographical structure based on 1,000 random permutations (Pons and Petit, 1996). *N*_ST_ > > *G*_ST_ strongly supported the presence of phylogeographical structure. The levels of variation within and among populations, and population differentiation statistics (*F*_ST_) values (Wright’s fixation index) were calculated by analyses of molecular variance (AMOVA) in Arlequin v. 3.1 (Excoffier and Lischer, 2010).^9^ Significance was tested using 10,000 permutations.

### Population Divergence and Dynamic Analyses

Divergence times estimation of the cpDNA haplotypes lineages were performed using BEAST v. 1.8 (Drummond et al., 2012).^10^ We downloaded the same chloroplast sequences from GenBank for *L. cestroides* (FJ189707, FJ189609) and *Atropa belladonna* (NC004561) species. *Lycium dasystemum*, *L. cestroides*, and *A. belladonna* served as the outgroups. An uncorrelated lognormal relaxed clock model with a Yule process was used for the speciation model. The GTR + I model was selected by jModelTest v. 2.1.5 (Darriba et al., 2019) and was used as the substitution model.^11^ As no fossil records were available for *Lycium*, each node was constrained by using the divergence times of *L. ruthenicum* and *L. dasystemum* (2.16 Mya) and *L. dasystemum* and *L. cestroides* (4.85 Mya) as secondary calibration points (Särkinen et al., 2013). For each BEAST analysis, Markov Chain Monte Carlo (MCMC) runs were performed on each of 1.0 × 10^7^ generations with sampling every 1,000 generations. The estimates and the convergence of the effective sample sizes (ESS; >200) for all parameters were tested by Tracer v. 1.5 (Drummond et al., 2012).^12^ A maximum clade credibility tree was compiled with Tree Annotator v. 1.7.5^13^ (Drummond et al., 2012) and FigTree v. 1.3.1^14^ was used to check the result.

The selective neutrality indices Tajima’s *D* (Tajima, 1989) and Fu’s *F_S_* (Fu, 1997) were estimated to detect *L. ruthenicum* population growth and expansion. A mismatch distribution analysis (MDA) was applied to measure population expansion for all samples. All expansion tests were implemented in Arlequin v. 3.1 (Excoffier and Lischer, 2010) which disclosed any evidence of recent demographic expansion. Unimodal pairwise distributions indicated demographic population expansions whereas stable populations exhibited bimodal or multimodal distributions. Goodness-of-fit was tested with the sum of squared deviation (*SSD*) between the observed and expected mismatch distributions as well as Harpending’s raggedness index (*H*_Rag_; Rogers and Harpending, 1992) using 1,000 parametric bootstrap replicates. When an expansion event was confirmed, the expansion time was calculated using the formula *T* = *τ*/2*μkg* (Rogers and Harpending, 1992) where *μ* is the substitution rate in substitutions/site/year (s/s/y) units, *k* is the average sequence length used for the analyses (*L. ruthenicum*: 1,110 bp), and *g* is the generation time in years (*g* = 2 for *L. ruthenicum*; Chen et al., 2014). For *L. ruthenicum*, the average cpDNA mutation rate (*μ*) set to 3.0 × 10^−9^ s/s/y was used for the estimation (Wolfe et al., 1987). The changes in effective population size for the cpDNA sequences were calculated using Bayesian skyline plots (BSP) in BEAST2 v. 2.6^15^ (Bouckaert et al., 2014) and the foregoing cpDNA sequence substitution parameters. For the cpDNA sequences, the MCMC chains were run for 5.0 × 10^7^ generations under the GTR + I substitution model. Both the MCMC convergence and the ESS (>200) were tested using Tracer v. 1.5.

### Potential Distribution Modeling

Ecological niche modeling was conducted using MAXENT v. 3.4.4^16^ (Phillips et al., 2006) to predict species occurrence under the last interglacial (LIG; *ca*. 0.13–0.14 Mya BP), the late-glacial maximum (LGM; *ca*. 0.02 Mya BP), the Mid-Holocene (MID; *ca*. 0.006 Mya BP), and current conditions (1970–2000). It was based on 7 years (2013–2019) of geographical distribution records for the *L. ruthenicum* populations included in the present study (Table 1) as well as those from the Chinese Virtual Herbarium,^17^ the Global Biodiversity Information Facility,^18^ previously published papers, and additional surveys during 2016–2019 (Chen et al., 2014, 2017). The number of location records of species within 12 km from other location records was removed to reduce the effects of spatial autocorrelation. Finally, 69 points were filtered from 113 distribution points for subsequent analysis (Supplementary Table S5), which was completed by using the ArcMap v. 10.6 (ESRI, Redlands, CA, United States). The Community Climate System Model (CCSM)^19^ was used to predict LGM and MID. Nineteen bioclimatic variables were obtained from the WorldClim database (Hijmans et al., 2005) at a resolution of 2.5 arcmin.^20^

SPSS v. 13.0 (IBM Corp., Armonk, NY, United States) was used to select eight climate factors with Pearson’s correlation coefficient < 0.8 for the subsequent modeling (Khanum et al., 2013; Promnun et al., 2021). The parameters chosen included (3) minimum temperature in the coldest month, (4) mean temperature in the driest quarter, (6) precipitation in the driest month, (9) mean temperature in the driest quarter, (10) mean temperature in the warmest quarter, (15) precipitation seasonality, (18) precipitation in the warmest quarter, and (19) precipitation in the coldest quarter. The model was generated with a randomly selected 75% training data, and the remaining 25% of the data was used for testing. Then, model validation was performed on 20 independent replicates, and other parameters with the default setting. The accuracy of each model prediction was tested by running a receiver operating characteristic (ROC) curve (Fawcett, 2006) analysis in MAXENT v. 3.4.4. Areas under the ROC curve (AUC) > 0.9 indicated good model fit (Swets, 1988).

## Results

### Microsatellite Diversity and Population Structure

The characteristics of 11 polymorphic microsatellite loci were identified in 540 *L. ruthenicum* individuals. The average number of alleles (*N*_A_), allelic richness (*A*_R_), observed heterozygosity (*H*_O_), expected heterozygosity (*H*_E_), and inbreeding coefficient (*F*_IS_) values are listed in Table 1. The mean species-range *N*_A_, *A*_R_, *H*_O_, *H*_E_, and *F*_IS_ were 28.975, 1.357, 0.384, 0.359, and − 0.053, respectively. The highest genetic diversity was found in the AL (Alashan) population (*N*_A_ = 44; *A*_R_ = 1.547; *H*_O_ = 0.264; *H*_E_ = 0.530) located in the northern parts of the Tianshan Mountains. AL was followed by GS (Minqin; *N*_A_ = 19; *A*_R_ = 1.451; *H*_O_ = 0.468; *H*_E_ = 0.346) and SC (Yarkant; *N*_A_ = 16; *A*_R_ = 1.432; *H*_O_ = 0.467; *H*_E_ = 0.372) populations located in the eastern and southern proportions of the range of the species (namely, the areas around the Qilian Mountains, the Qaidam Basin, and north of the Kunlun Mountains), respectively.

**Figure 1:**
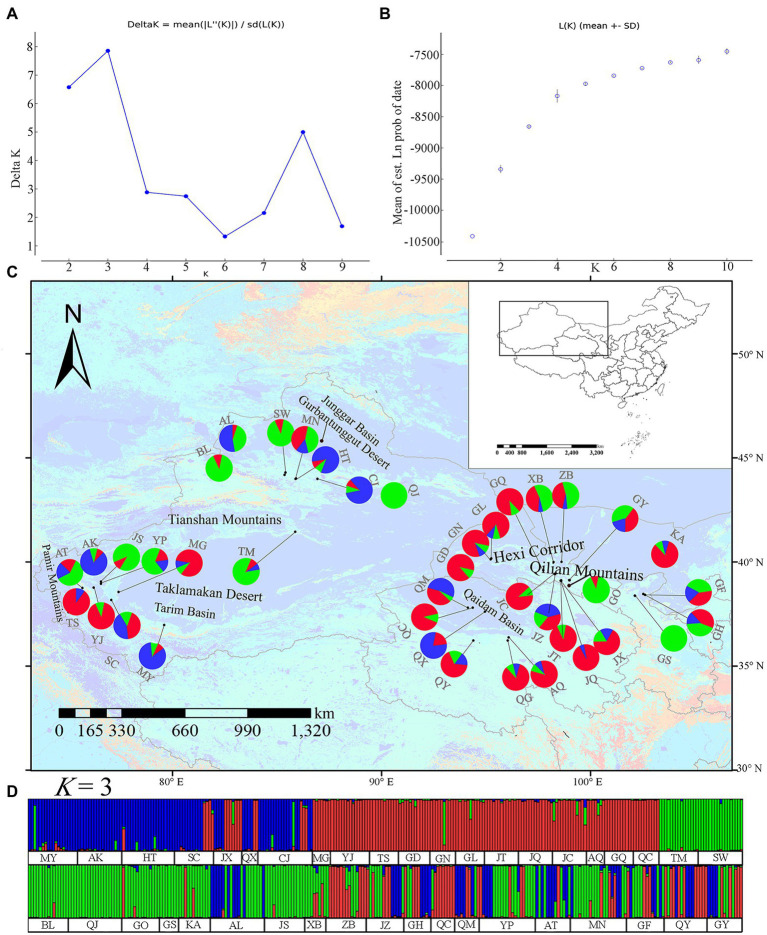
Genetic divergence in 40 *Lycium ruthenicum* populations based on 11 EST-SSR datasets. **(A)** Log-likelihood values. Ln P (*K*) as a function of *K* calculated for 10 replicates. **(B)** Second-order changes in the log-likelihood values. (Delta *K*) as a function of *K* calculated for 10 replicates. **(C)** Geographical distribution of three genetic clusters (*K* = 3) and genetic cluster composition in each population. **(D)** Proportion of genetic clusters at *K* = 3 for each of 540 individuals. Smallest vertical bars represent individuals.

The STRUCTURE analysis results indicate that ∆*K* reached its maximum value at *K* = 3 (Figures 1A,B). Though we selected *K* = 3 for the final analysis, the clusters were not geographically associated. Therefore, the STRUCTURE analysis did not detect any geographically meaningful genetic cluster (Figures 1C,D).

### Chloroplast Haplotype Variation and Distribution

**Figure 2:**
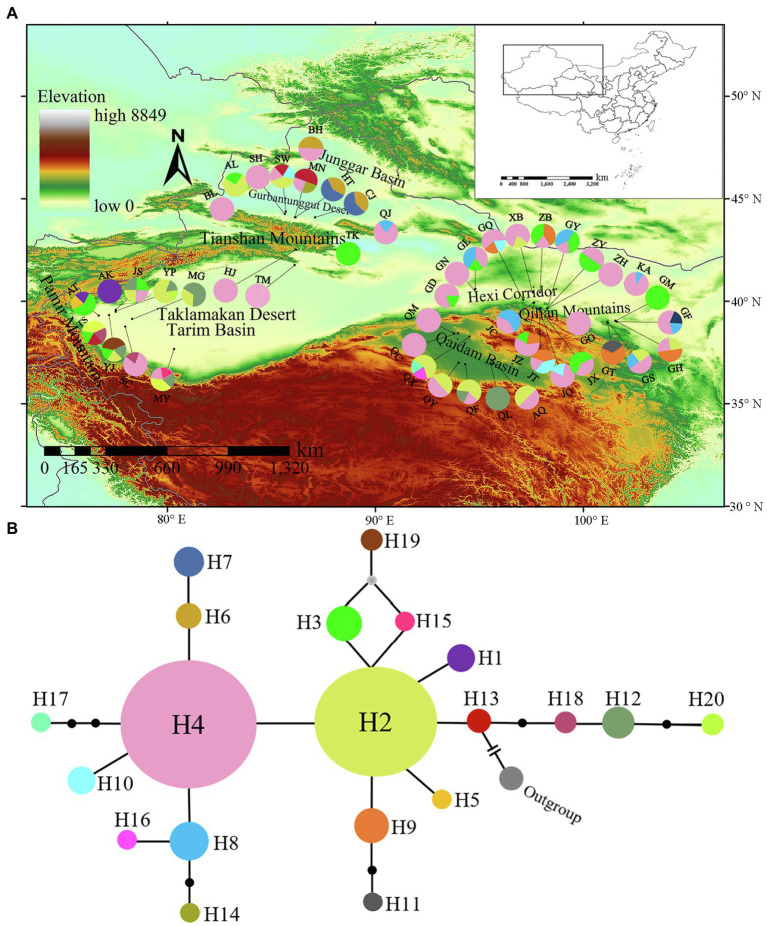
Analysis of cpDNA fragment (*rps16*–*trnK* and *trnH*–*psbA*) haplotypes of *Lycium ruthenicum*. **(A)** Geographical distributions of cpDNA haplotypes. **(B)** Statistical parsimony network of cpDNA haplotypes. Circle sizes are roughly proportional to individual numbers (*n*) of haplotypes. Smallest circles represent *n* = 1 while largest circles represent *n* = 88. Solid lines represent mutational steps interconnecting two haplotypes. Each line represents 1 step.

Alignment of the cpDNA sequences for 206 *L. ruthenicum* individuals yielded a consensus sequence 1,110 bp long, namely, 728 bp for *rps16*–*trnK* and 382 bp for *trnH*–*psbA*, respectively. Twenty haplotypes were identified in 49 populations. There were 20 variable sites, 13 nucleotide substitutions, six indels, and one inversion 29 bp long in the *trnH*–*psbA* region (Supplementary Table S4). Haplotypes H2 and H4 had the widest distributions followed by H3. Haplotypes H5, H11, H14, H15, H16, H17, H19, and H20 were observed in the AT (Atushi), GT (Minqin), MN (Manas), MY (Moyu), QX (Haixi), QX, YJ (Yengisar), and YP (Yopurga) populations, respectively (see Supplementary Table S1 for more details). H4, H2, and H3 were detected in 88, 31, and 23 samples, respectively. They were the dominant haplotypes and widely distributed in the Tarim Basin, the Junggar Basin, the Hexi Corridor, the Qaidam Basin, Ningxia, and parts of Inner Mongolia (Table 1; Figure 2A).

A median-joining network analysis was used to determine the relationships among the cpDNA haplotypes (Figure 2B). The haplotypes relationships revealed that H13 was the ancestral haplotype. It was distributed in the northern Tianshan Mountains (Manas: MN, Shawan: SW) and the eastern Pamir Mountains (Tashkurgan: TS; Figures 2A,B).

### Genetic Diversity and Population Genetic Structure

Among all populations, the species-level cpDNA haplotype and nucleotide diversity values were *h* = 0.776 and *Pi* = 0.00152, respectively (Table 1). The foregoing results indicated that the *L. ruthenicum* populations had high species-level haplotype diversity (*h* = 0.776) and low species-level nucleotide diversity (*Pi* = 0.00152). The phylogeographic structure between haplotypes did not significantly contribute to population differentiation (*G*_ST_ = 0.308; *N*_ST_ = 0.226; *G*_ST_ > *N*_ST_; *p* > 0.05). Hence, the species had no significant phylogeographical structure. The results of AMOVA showed that a large proportion of the variation (65.64%) occurred within population; this result was consistent with the high genetic differentiation within population of this species (*F*_ST_ = 0.35; *p* < 0.001; Table 2).

**Table 2:** Results of analysis of molecular variance (AMOVA) of *Lycium ruthenicum* populations.

Source of variation	*df*	Sum of squares	Variance components	Percentage of variation	*p*	Fixation index
Among populations	49	89.449	0.303	34.36	*p* < 0.001	
Within populations	156	90.420	0.580	65.64	*p* < 0.001	*F*_ST_ = 0.3504

*df*, degrees of freedom.

### Demographic History and Divergence Times Estimation

**Figure 3:**
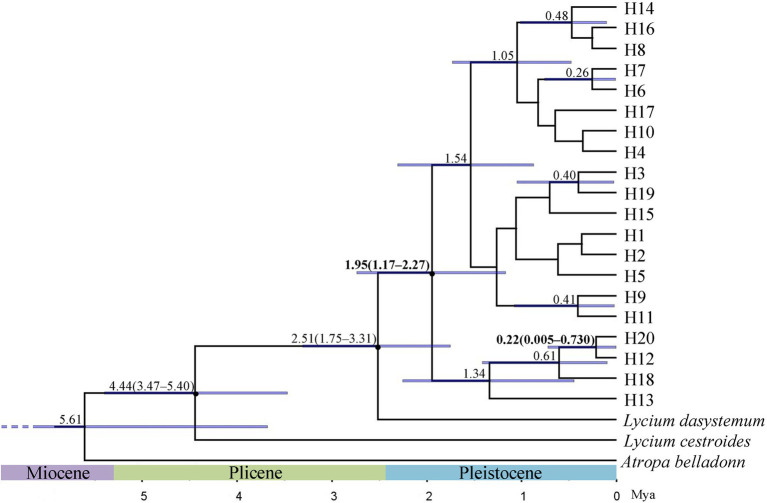
Phylogenetic tree of 20 haplotypes constructed by Bayesian analysis of *Lycium ruthenicum*. Divergence times (millions of years ago; Mya) of nodes with 95% ranges of highest posterior density (95% HPD) are shown above branches. Blue horizontal bars indicate 95% HPD of node age.

BEAST was used to estimate the divergence times. When *L. dasystemum*, *L. cestroides*, and *A. belladonna* were used as outgroups, the phylogenetic tree showed that all *L. ruthenicum* haplotypes formed a monophyletic group. It was estimated that the differentiation times among these 20 haplotypes ranged from the early Pleistocene [1.95 Mya; 95% highest posterior density (HPD) = 1.17–2.74 Mya] to the middle Pleistocene (0.22 Mya; 95% HPD = 0.005–0.730 Mya; Figure 3).

**Figure 4:**
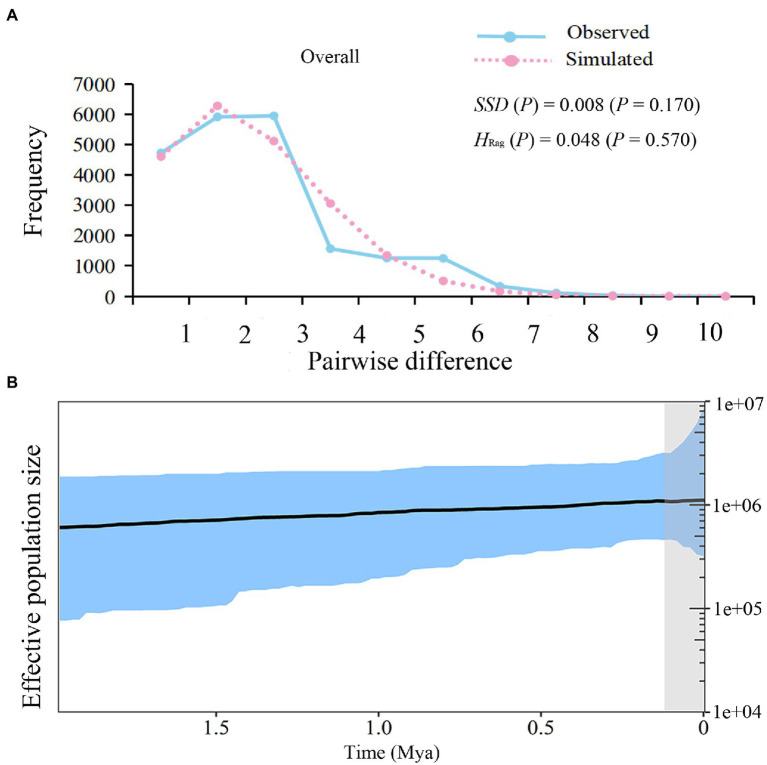
Demographic Analysis of *Lycium ruthenicum*. **(A)** Pairwise mismatch distributions of cpDNA sequences. **(B)** Bayesian skyline plot (BSP) based on cpDNA for effective population size fluctuation over time. Black line is median estimation. Area between blue lines is 95% CI.

For all *L. ruthenicum* groups, the neutrality test statistics revealed significantly negative Tajima’s *D* (*D* = −1.443; *p* = 0.037) and Fu’s *F*_S_ (*F*_S_ = −8.877; *p* = 0.009). The unimodal mismatch distribution, the positive *SSD* values (*SSD* = 0.008; *p* = 0.170), and the *H*_Rag_ (0.048; *p* = 0.570; Table 3; Figure 4A) consistently suggested a past demographic expansion event.

**Table 3:** Results of neutrality tests and mismatch distribution analysis for *Lycium ruthenicum* populations.

Group	*τ*	Expansion time (t,Ma)	*SSD* (*p*)	*H*_Rag_ (*p*)	Tajima’s *D* (*p*)	Fu’s *F_S_* (*p*)
Overall	1.70	0.12	0.008 (0.170)	0.048 (0.570)	−1.443 (0.037)	−8.877 (0.009)

H_Rag_, the Harpending’s raggedness index; SSD, sum of squared deviations; and *τ*: range expansion parameter.

The unimodal mismatch distribution and relatively low *SSD* and *H*_Rag_ values indicated past species-level expansion events. The estimated expansion time was *ca*. 0.12 Mya. A Bayesian skyline plot (BSP) displayed a steady growth between *ca*. 1.95 and 0.12 Mya while the species-level rapid expansion events occurred since the past *ca*. 0.12 Mya (Figure 4B).

### Ecological Niche Modeling

**Figure 5:**
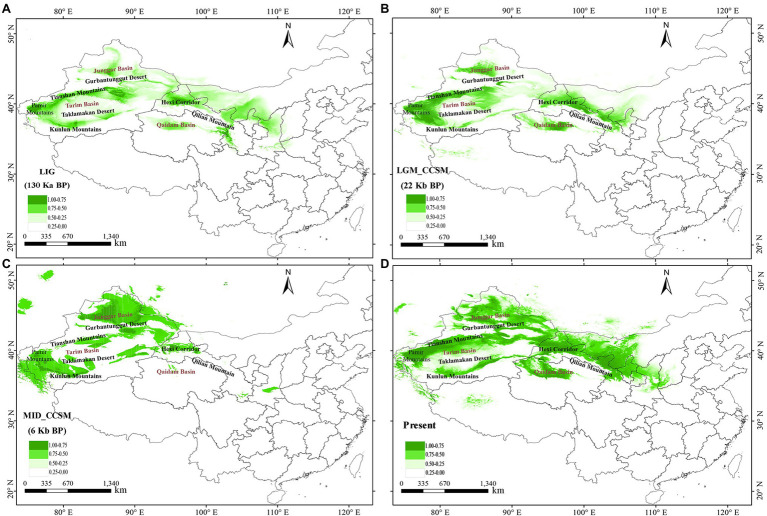
Model of areas climatically suitable for *Lycium ruthenicum* at different times. **(A)** Last interglacial (LIG; *ca*. 0.13–0.14 Mya BP). **(B)** Last glacial maximum (LGM; *ca*. 0.02 Mya BP) under Community Climate System Model (CCSM). **(C)** Mid Holocene (MID; *ca*. 0.006 Mya BP) under CCSM. **(D)** Present time (1970–2000).

The AUC values for *L. ruthenicum* ENM were > 0.90 for all four periods. The predictive model performed well. The predicted distribution of the species under current conditions is generally similar to its actual distribution in northwestern China. The suitable habitats of the *L. ruthenicum* populations were narrower and more fragmented during the Last Interglacial (LIG) and Late Glacial Maximum (LGM) than they are at present (Figures 5A,B). The ENM predicted that the potential distribution areas in LIG and LGM were near the Junggar Basin, the Pamir Plateau, the western part of Tarim Basin, the Qaidam Basin, and the Hexi Corridor, and the small areas of the Tianshan Mountains. Interestingly, however, the distribution range of *L. ruthenicum* in the LIG period seems to have been less than the LGM (Figures 5A,B). The MID and current distribution areas of *L. ruthenicum* greatly expanded near the Pamir Plateau and in the Tianshan Mountains, Tarim Basin, Junggar Basin, the Qaidam Basin, and Hexi Corridor, and parts of Inner Mongolia (Figures 5C,D).

## Discussion

### Genetic Diversity and Genetic Structure

The two chloroplast intergenic fragments *rps16*–*trnK* and *trnH*–*psbA* indicated high species-level haplotype diversity (*h* = 0.776) but low species-level nucleotide diversity (*Pi* = 0.00152) for *L. ruthenicum* (Table 1). A previous cpDNA analysis reported similar results (Chen et al., 2014). During the long evolutionary process of species expansion, new haplotypes accumulated through mutations whereas nucleotide sequence diversity did not (Avise, 2000). This phenomenon may be explained by the low nucleotide substitution rate and highly conserved genomic structure of the chloroplast genome (Daniell et al., 2016). Similar results were reported for the desert plants *Lagochilus ilicifolius* (*h* = 0.8824, *Pi* = 0.0016; Meng and Zhang, 2011) and *Zygophyllum xanthoxylon* (*h* = 0.535, *Pi* = 0.0049; Shi and Zhang, 2015) in northwestern China. AMOVA demonstrated that a large proportion of the variations occurred within populations (Table 2). In contrast, the genetic variation among populations was relatively low. Thus, there was a high level of gene flow among populations. The opposite results were reported for other shrub species in northwestern China such as *Lagochilus* (Meng and Zhang, 2013) and *Gymnocarpos przewalskii* (Ma et al., 2012).

The chloroplast and microsatellite marker assays did not significantly differentiate among *L. ruthenicum* populations (Figures 1, 2). Previous SRAP and ISSR analyses reported similar observations (Liu et al., 2012b; Alitong et al., 2013). The reasons for the lack of phylogeographical structure in *L. ruthenicum* may be complex. Firstly, *L. ruthenicum* outcrosses and has partial self-incompatibility (Hitchcock, 1932; Savage and Miller, 2006; Kuang and Lu, 2011). Outcrossing usually enhances progeny viability and establishes and maintains high overall genetic diversity in plants (Hamrick, 1982; Borba and Shepherd, 2001). The pollination of *L. ruthenicum* is mediated by wind and insects (Hedrick, 2004), and the small seeds of *L. ruthenicum* are contained in fleshy, bright black fruits and are spread by the birds and rodents that ingest them (Hitchcock, 1932). *Lycium ruthenicum* seeds mature in mid-October and are dispersed to deserts *via* wind and dust storm (Liu et al., 2012b). Secondly, *Lycium ruthenicum* is salt- and drought-tolerant, most individuals are long-lived, and the root of *L. ruthenicum* is propagated *via* clones (Dong et al., 2008; Dai et al., 2013). Finally, the geographical distribution of a species may also influence its genetic diversity. As a rule, genetic variation is comparatively higher in widely distributed species (Hamrick and Godt, 1996). The abovementioned factors might explain the high genetic diversity of *L. ruthenicum*.

### Potential Glacial Refugia

Ice sheets did not cover most of northwestern China, but significant Quaternary climatic oscillations nonetheless occurred (Meng et al., 2015). During the Pleistocene glaciations, extreme drought and low temperatures fragmented desert plant habitats. However, the warmer, wetter conditions of the interglacial period might have promoted desert plant expansion. Nevertheless, the ENM estimated that both the LIG and LGM (Figures 5A,B) climatic fluctuations might have substantially narrowed the range of this desert species compared to its current range. Thus, we propose that the relatively drought and temperatures conditions of the LIG period (*ca*. 0.13–0.14 Mya) were not conducive to the expansion of *L. ruthenicum*. For this reason, this species retreated to the refugia situated on the edges of the Junggar Basin, the Tarim Basin, the Tianshan Mountains, the Pamir Plateau, the Qaidam Basin, and near the Qilian Mountains during the LIG and LGM (Figures 5A,B). Similar expansion patterns were found in another desert plant, *Populus euphratica* (Zeng et al., 2018).

During the Pleistocene, drastic climatic fluctuation caused the migration and extinction of large plants. Harsh cold and dry climates caused them to retreat to refugia (Hewitt, 2000; Shen et al., 2002). These refugia also became starting points for rapid range expansion and recolonization of species after the glacial periods (Comes and Kadereit, 1998; Taberlet et al., 1998; Liu et al., 2012a). Glacial refugia have unique geographical conditions and are suitable living environments. Therefore, they harbor high levels of genetic diversity and ancestral and unique haplotypes. The Junggar Basin, the Tianshan Mountains, the western Tarim Basin, the eastern Pamir Plateau, the Qaidam Basin, and the Hexi Corridor are glacial refugia for certain desert plants in northwestern China (Zhang and Zhang, 2012; Zhang et al., 2016).

Our ENM predicted that the potential distribution areas of *L. ruthenicum* in the LIG (Figure 5A) were located near the Junggar Basin, the Pamir Plateau, and the Hexi Corridor whereas those during the LGM (Figure 5B) were mainly localized to the western edge of the Tarim Basin, the Junggar Basin and the northern part of the Tianshan Mountains, the Qaidam Basin, and the Hexi Corridor. Thus, based on ancestral haplotype distribution and ENM analysis results, we hypothesize that *L. ruthenicum* had two possible refugia during the Pleistocene Period of which one was situated in the southern part of the Junggar Basin and the northern part of the Tianshan Mountains. The genetic diversity of the northern populations in this area (*N_A_* = 29.286; *A_R_* = 1.381; *H_O_* = 0.394; *H_E_* = 0.401; Table 1) was higher than those of the southern and eastern populations. Moreover, the populations located in the southern parts of the Junggar Basin and the northern part of the Tianshan Mountains included one ancestral haplotype (H13 in the MN and SW populations) and one unique haplotype (H6 in the CJ (Changji), BH (Habahe), and HT (Hutubi) populations; Table 1). The northern branches of the Tianshan Mountains and the Junggar Basin comprise a biodiversity hotspot in northwestern China (Meng et al., 2015). This region was relatively wet and a suitable environment for desert plants during the glacial period. Hence, it was also a potential refugium of *L. ruthenicum*. The results align with those reported for phylogeographical studies on the desert plants *Capparis spinosa* (Wang et al., 2016b) and *Zygophyllum xanthoxylon* (Shi and Zhang, 2015).

Other putative refugia are located in the western Tarim Basin and the eastern Pamir Plateau. In these regions, there was one ancestral haplotype (H13 in the TS population) and five unique haplotypes (H5, H15, H18, H19, and H20 in the AT, MY, SC and TS, YJ, and YP populations, respectively; Table 1). The western areas of the Tarim Basin and the eastern areas of the Pamir Plateau were supplied with meltwater from the snow and glacial ice on the mountains (Meng et al., 2015). Therefore, this area was conducive to *L. ruthenicum*’s survival. These putative refugia were identified in a previous study (Ma et al., 2012).

### Demographic History of *Lycium ruthenicum* in the Quaternary

Significantly (*p* < 0.05) negative values for the neutrality test statistics, unimodal mismatch distribution, and relatively low *SSD* and *H*_Rag_ (*p* > 0.05; Table 3; Figure 4A) are all indicative of a past expansion event in this species (Hudson, 1990). The existence of widespread haplotypes (H2, H4), other star-like patterns of the haplotype network (Figure 2B), and the BSP (Figure 4B) are all evidence of the demographic expansion of *L. ruthenicum* (Hudson, 1990).

The rapid uplift of the Tianshan and other mountain ranges related to the QTP after *ca*. 2.6 Mya dramatically hindered local precipitation and hydrological circulation and intensified the aridification in northwestern China (Harrison et al., 1992; Spicer et al., 2003; Liu et al., 2014a). Simultaneous monsoon intensification might have had further enhanced the climatic aridification (Su et al., 2011). The BSP result showed that *L. ruthenicum* underwent rapid expansion over the past *ca*. 0.12 Mya (Figure 4B). The suitable habitats of the *L. ruthenicum* populations were narrower and more fragmented during the LIG (*ca*. 0.13–0.14 Mya) and LGM (*ca*. 0.02 Mya; Figures 5A,B). A study reported that northwestern China entered its largest glacial period at *ca*. 1.2–0.6 Mya (Cun and Wang, 2010). Furthermore, the Gonghe movement occurred during the past *ca*. 0.14 Mya changed the climate and intensified aridification in northwestern China. Therefore, the geological and climatic aridification of the LIG (Figure 5A) might have substantially narrowed the range of this desert species. During the LGM (Figure 5B), cold and dry climates caused the *L. ruthenicum* species to retreat to the edges of the Tarim Basin, the Junggar Basin, the Qaidam Basin, and the Hexi Corridor. Thus, the suitable habitats of the *L. ruthenicum* populations were narrower and more fragmented during the LIG and LGM than they are at present (Figure 5D).

Several recent studies reported that many arid land plants in northwestern China are more inclined to migrate along the edges of deserts during the interglacial periods (Xu and Zhang, 2015). During the late Pleistocene (*ca*. 0.126–0.012 Mya), the climate of northwestern China has fluctuated between humid and dry conditions, resulting in the enlargement of deserts while the desert margin (e.g., Taklamakan Desert of the Tarim Basin and Gurbantunggut Desert of the Junggar Basin, Badain Jaran–Tengger desert to the north of the Hexi Corridor) and arid piedmont grassland areas seem to have provided stable habitats for desert plants (Su and Zhang, 2014). Although *L. ruthenicum* is generally adapted to drought, its habitats do not occur within the climatically extreme desert interior. Thus, the relatively humid desert margin areas seem to provide relatively stable habitats for this species (Dai et al., 2013). Subsequently, during the early Holocene (*ca*. 0.01 Mya) period, snow and glacial ice melted off the Tianshan, the Altai, the Kunlun Mountains, and Qilian Mountains surrounding the Junggar, Tarim Basins, and Qaidam Basin irrigating the surrounding deserts and increased the humidity on the edge of these regions (Yang and Scuderi, 2010; Meng et al., 2015). It is, therefore, possible that under these warm conditions the *L. ruthenicum* species could thrive and expand outwards along the edges of the Gurbantunggut and Taklamakan Deserts and migrated westward *via* the Hexi Corridor. These migration patterns are consistent with putative colonization routes in Hexi Corridor proposed by Meng et al. (2015) in the review about the plant phylogeography in arid northwestern China, and similar results have been found in other desert plants, e.g., *Zygophyllum xanthoxylon* (Shi and Zhang, 2015). The foregoing results support the hypothesis that under warm climate conditions following the LGM period (Figure 5B), the geographical range of *L. ruthenicum* greatly increased and the species became widely distributed in northwestern China. This postulate aligns with the present distribution pattern of *L. ruthenicum* in this region. Our results were similar to those reported for other desert plants such as *Hexinia polydichotoma* (Su and Zhang, 2014), and *Reaumuria soongarica* (Yin et al., 2015). Nevertheless, the results support that compared with alpine plants, the response of drought-tolerant desert plants to the geology and climatic oscillations in the Pleistocene might not be consistent with the expansion and contraction of high latitude ice sheets, but rather, the expansion of species range mainly depended on the temperature and relative humidity on the edge of the desert and adjacent areas (Meng and Zhang, 2013; Meng et al., 2015).

### Conservation Insights

Genetic diversity is vital for a species to be able to maintain its life cycle, reproduce, resist disease, and adapt to changing environmental conditions (Avise and Hamrick, 1996). The conservation of desert plant genetic resources is essential to mitigate any further degradation of fragile arid and semi-arid ecosystems and maintain desert biodiversity. *Lycium ruthenicum* is an important medicinal plant with high economic and ecological value. For this reason, the protection of this wild resource is imperative.

Populations with high genetic diversity should be prioritized for conservation measures. Here, the northern part of the Tianshan Mountains had higher genetic diversity than the eastern and southern regions within the range of *L. ruthenicum*. Thus, the northern populations of this species require protection. The refugial populations MN, SW, CJ, and BH are at particularly high risk and merit conservation. The southern refugial populations TS, AT, MN, MY, SC, YJ, and YP in the western Tarim Basin and eastern Pamir Plateau also need protection. Conservation of these populations is crucial for the preservation of the genetic diversity of this species in the face of future climate change. We propose that cultivated *L. ruthenicum* should substitute for wild resources in commercial applications and a horticultural system should be established to protect existing wild populations.

## Conclusion

The present study clarified the influence of climatic oscillations events upon the geographical distribution and the demographic history of the salt- and drought-tolerant desert plant *Lycium ruthenicum* in the Quaternary. By combining the evidence from both ENM and molecular data, we first confirmed that the present spatial genetic structure of *L. ruthenicum* in northwestern China resulted from the combined effects of expansion and contraction from refugia after the LGM. This event was predominantly driven by the Pleistocene extreme drought and low temperatures resulting from the rapid uplifting of QTP and other mountains. The evidence indicated an overall consensus between the present geographical distribution of *L. ruthenicum* and the effects of the geological events, climatic fluctuations, and aridification during the Quaternary in northwestern China. The two molecular data assays indicated that *L. ruthenicum* had high genetic diversity and there was no significant differentiation among its populations. Hence, we propose that breeding systems, long-term seed dispersal, and postglacial range expansion may have increased the genetic diversity and diluted the regional differentiation of this species. In addition, protecting the natural wild plant populations of *L. ruthenicum* will help to mitigate wind erosion, and maintain the integrity of the desert ecosystem in the study region. These findings have implications for thinking about the refugia of other drought-tolerant desert plants in northwestern China as well as provide new insight into the demographic expansion of plants and maintaining high species diversity in this arid region.

## Data Availability Statement

The data presented in the study are deposited in the National Center for Biotechnology Information (NCBI) repository, accession numbers, ON055439, ON055440, ON390854–ON390893.

## Author Contributions

JL, PL, and X-MT conceived the ideas. GY and JL contributed to the sampling. GY performed the experiments and analyzed the data. GY, PL, and X-MT wrote the manuscript. C-XW, M-QX, and HC revised the manuscript and provided useful suggestions. All authors contributed to the article and approved the submitted version.

## Funding

This research was supported by the National Natural Science Foundation of China (grant nos. 31970225 and 31760102), the Zhejiang Provincial Natural Science Foundation (LY19C030007), the “Xinjiang Key Laboratory of Special Species Conservation and Regulatory Biology,” and the “13th Five-Year” Plan for Key Discipline Biology, Xinjiang Normal University.

## Conflict of Interest

The authors declare that the research was conducted in the absence of any commercial or financial relationships that could be construed as a potential conflict of interest.

## Publisher’s Note

All claims expressed in this article are solely those of the authors and do not necessarily represent those of their affiliated organizations, or those of the publisher, the editors and the reviewers. Any product that may be evaluated in this article, or claim that may be made by its manufacturer, is not guaranteed or endorsed by the publisher.

## Footnotes

^1^ http://www.gov.cn/zhengce/zhengceku/2021-09/09/content_5636409.htm

^2^ https://www2.unil.ch/popgen/softwares/fstat.htm

^3^ https://web.stanford.edu/group/pritchardlab/structure_software/release_versions/v2.3.3/html/structure.html

^4^ http://taylor0.biology.ucla.edu/structureHarvester/

^5^ https://www.geneious.com/

^6^ https://mafft.cbrc.jp/alignment/software

^7^ http://www.ub.edu/dnasp/index_v5.html

^8^ http://www.fluxus-engineering.com

^9^ http://cmpg.unibe.ch/software/arlequin3/

^10^ https://beast.community/2016-06-17_BEAST_v1.8.4_released.html

^11^ https://github.com/ddarriba/jmodeltest2/release/

^12^ https://github.com/beast-dev/tracer/

^13^ https://github.com/krmanik/Anki-xiehanzi/tree/v1.7.5

^14^ https://figtree-1-3-1.software.informer.com/1.3/

^15^ https://github.com/CompEvol/beast2/release/

^16^ https://biodiversityinformatics.amnh.org/open_source/maxent/

^17^ http://www.cvh.ac.cn/

^18^ https://www.gbif.org

^19^ https://www.cesm.ucar.edu/models/ccsm4.0/

^20^ http://www.worldclim.org
