# Supplementary material for: Phylogeography and Population Genetics Analyses Reveal Evolutionary History of the Desert Resource Plant Lycium ruthenicum (Solanaceae)
Source: Front Plant Sci. 2022 Jun 30;13:915526. doi: 10.3389/fpls.2022.915526 (PMC9280156; doi:10.3389/fpls.2022.915526)
Supplement: Supplementary file 1 [file Data_Sheet_1.docx]

**Table S1** Sampling information of *Lycium ruthenicum* populations in the present study

| Population code | Geographic site | Latitude (N) | Longitude (E) |
| --- | --- | --- | --- |
| AQ | Wutu, Nuomuhong, Doulan, haixi, Qinghai | 37°00′ | 96°51′ |
| GD | Changfeng, Huangqu, Dunhuang, Jiuquan, Gansu | 40°13′ | 95°11′ |
| GF | Jingyuan, Baiyin, Gansu | 39°10′ | 103°03′ |
| GH | Minqin, Wuwei, Gansu | 39°05′ | 103°12′ |
| GL | Guazhou, Jiuquan, Gansu | 40°38′ | 96°04′ |
| GM | Minqin, Wuwei, Gansu | 39°11′ | 103°03′ |
| GN | Nancha, Guazhou, Jiuquan, Gansu | 40°43′ | 96°07′ |
| GO | Pujiazhuang, Ganzhou, Zhangye, Gansu | 39°26′ | 100°59′ |
| GQ | Yuquan, Jiayuguan, Gansu | 40°25′ | 98°18′ |
| GS | Minqin, Wuwei, Gansu | 39°02′ | 103°03′ |
| GT | Minqin, Wuwei, Gansu | 39°03′ | 103°02′ |
| GY | Sunan, Zhangye, Gansu | 39°38′ | 99°44′ |
| JC | Suzhou, Jiuquan, Gansu | 40°08′ | 99°13 |
| JQ | Suzhou, Jiuquan, Gansu | 39°37′ | 99°05′ |
| JT | Suzhou, Jiuquan, Gansu | 39°36′ | 99°03′ |
| JX | Suzhou, Jiuquan, Gansu | 39°34′ | 99°05′ |
| JZ | Suzhou, Jiuquan, Gansu | 40°08′ | 99°16′ |
| KA | Alxa, Inner Mongolia | 41°15′ | 103°54′ |
| QC | Dachaidan, Haixi, Qinghai | 38°06′ | 95°18′ |
| QF | Greermu, Qinghai | 36°37′ | 95°14′ |
| QG | Dulan, Haixi, Qinghai | 36°39′ | 96°46′ |
| QL | Dulan, Haixi, Qinghai | 36°45′ | 96°47′ |
| QM | Dachaidan, Haixi, Qinghai | 38°05′ | 94°53′ |
| QX | Dachaidan, Haixi, Qinghai | 37°25′ | 95°36′ |
| QY | Greermu, Qinghai | 36°46′ | 95°20′ |
| XB | Xiba, Jinta, Jiuquan, Gansu | 40°25′ | 99°09′ |
| ZB | Jinta, Jiuquan, Gansu | 40°26′ | 99°07′ |
| ZH | Sunan, Zhangye, Gansu | 39°38′ | 99°47′ |
| ZY | Sunan, Zhangye, Gansu | 39°36′ | 99°45′ |
| AK | Akto, Kizilsu, Xinjiang | 39°00′ | 76°26′ |
| AT | Atushi, Xinjiang | 40°15′ | 75°58′ |
| HJ | Hejing, Bayingolin, Xinjiang | 42°54′ | 86°24′ |
| JS | Payzawat, Kashgar, Xinjiang | 39°32′ | 77°16′ |
| MG | Makit, Kashgar, Xinjiang | 39°21′ | 78°02′ |
| MY | Moyu, Hetian, Xinjiang | 37°20′ | 80°23′ |
| SC | Yarkant, Kashgar, Xinjian | 38°41′ | 77°27′ |
| TM | Tiemenguan, Bayingolin, Xinjiang | 42°12′ | 86°17′ |
| TS | Tashkurgan, Kashgar, Xinjiang | 38°22′ | 75°20′ |
| YJ | Yengisar, Kashgar, Xinjiang | 39°01′ | 76°42′ |
| YP | Yopurga, Kashgar, Xinjiang | 39°21′ | 77°17′ |
| AL | Alashan, Bortala, Xinjiang | 85°52′ | 40°37′ |
| BH | Altay, Habahe, Xinjiang | 48°06′ | 86°43′ |
| BL | Bole, Bortala, Xinjiang | 45°10′ | 83°04′ |
| CJ | Changji, Xinjiang | 44°28′ | 87°25′ |
| HT | Hutubi, changji, Xinjiang | 44°29′ | 86°22′ |
| MN | Manas, changji, Xinjiang | 40°43′ | 86°18′ |
| QJ | Qi jiao jing, Hami, Xinjiang | 43°48′ | 91°48′ |
| SH | Shihezi, Xinjiang | 44°48′ | 86°07′ |
| SW | Shawan, Yili, Xinjiang | 45°00′ | 86°10′ |
| TK | Toksun, Turpan, Xinjiang | 43°20′ | 89°07′ |

**Table S****2** Characteristics of 11 microsatellite loci developed for *Lycium ruthenicum*

| Locus | Primer sequences (5’→3’) | Repeat motif | GenBank accession no | T_a_ (°C) |
| --- | --- | --- | --- | --- |
| U20 | F: CCCACTTCTCAAAAATGGTACAC | (ATT)6 | KY284848 | 58.3° |
|  | R: ATAGTTGCCAACAAACCCTTCTT |  |  |  |
| U21 | F: GGATGAAGAAGAAGAGGATGACA | (AAT)6 | KY284849 | 59° |
|  | R: CTTCTCAAAAATGGTACACTGCC |  |  |  |
| U23 | F: CTACTTCCATTTGTGGAAAGCTG | (TGC)6 | KY284850 | 58.3° |
|  | R: TAGCCAGTCTAATCTTCGGTTTG |  |  |  |
| U25 | F: CAGGAAGGAGAAGAGTCTGATGA | (GCA)5 | KY284851 | 58.3° |
|  | R: TTATCATTAACGGCTTCCATTTG |  |  |  |
| U26 | F: AATGGGGAAAGGTAAAGGAAGTT | (GTA)6 | KY284852 | 58.3° |
|  | R: CCTTGTGGAATTTTACTTTCCAAT |  |  |  |
| U27 | F: CCACCCAGATAGTGGTGGTAATA | (GAA)6 | KY284853 | 59° |
|  | R: GCTGATGTTTTCACATTTGTCAC |  |  |  |
| U31 | F: TAGGGTTTGAGGGTTTGAAGAAT | (CAC)5 | KY284854 | 59° |
|  | R: ATTATTATGGCTTCTTCACCTGG |  |  |  |
| U36 | F: CTACCACTCCAACGTGTACCAAT | (CAA)6 | KY284855 | 60° |
|  | R: TTCTTGCTCTAATTCTGAAACCG |  |  |  |
| U42 | F: GTCTCCATTTTACCCCTACCAAG | (ATT)6 | KY284856 | 60° |
|  | R:TTTGCAAATAAAATGCGATTATTG |  |  |  |
| U46 | F: ATGAAGGCAATATTTAGGGCAGT | (TTG)7 | KY284857 | 60° |
|  | R: CAATTTCATATTTGTGCTCTGCAT |  |  |  |
| D1 | F: TTCCAAGAACATTAGCACAAACA | (TTGGCT)4 | KY284858 | 59° |
|  | R: TGGCACTTGTCCTAGTCCTAAAC |  |  |  |

T_a_ = optimized annealing temperature.

**Table S3** PCR primers used to amplify the *Lycium ruthenicum* cpDNA fragment (*rps16*–*trnK* and *trnH*–*psbA*)

| Primer | Primer sequences (5’ → 3’) | References | Ta (°C) |
| --- | --- | --- | --- |
| *^3^’rps16*–*^5^’trnK* | F: TTA AAA GCC GAG TAC TCT ACC  R: AAA GTG GGT TTT TAT GAT CC | Shaw et al., 2005 | 51℃ |
| *TrnH*^GUG^–*rpsA* | F: CGCGCATGGTGGATTCACAATCC  R: GTTATGCATGAACGTAATGCTC | Shaw et al., 2007 | 57.5℃ |

T_a_ = optimized annealing temperature

**Table S4** Variable sites of each genotype for the cpDNA fragment (*rps16*–*trnK* and *trnH*–*psbA*) in *Lycium ruthenicum*

| Haplotype | *rps16-trnK* | | | | | | | | | | |  | *trnH-psbA* | | | | | | | | |
| --- | --- | --- | --- | --- | --- | --- | --- | --- | --- | --- | --- | --- | --- | --- | --- | --- | --- | --- | --- | --- | --- |
|  | 35 | 39 | 42 | 73 | 104 | 108 | 261 | 425 | 453 | 459 | 660 |  | 33 | 72 | 92 | 93 | 95 | 187 | 238 | 295 | 331 |
| H1 | C | A | T | A | A | C | T | G | A | C | A |  | G | A | A | C | C | C | T | A | a |
| H2 | **A** | A | T | A | A | C | T | G | A | C | A |  | G | A | A | C | C | C | T | A | a |
| H3 | **A** | A | T | A | A | C | T | G | A | C | **C** |  | G | A | A | C | C | C | T | A | a |
| H4 | **A** | A | T | A | A | C | T | G | A | C | A |  | G | A | A | C | C | **A** | T | A | a |
| H5 | **A** | A | T | A | A | C | T | **3** | A | C | A |  | G | A | A | C | C | C | T | A | a |
| H6 | **A** | A | T | A | A | C | T | G | A | C | A |  | **T** | A | A | C | C | **A** | T | A | a |
| H7 | **A** | A | T | **1** | A | C | T | G | A | C | A |  | **T** | A | A | C | C | **A** | T | A | a |
| H8 | **A** | A | T | A | A | C | T | G | A | C | A |  | G | A | A | C | C | **A** | **G** | A | a |
| H9 | **A** | A | T | A | A | C | T | G | A | C | A |  | G | A | A | C | C | C | T | A | **7** |
| H10 | **A** | A | T | A | A | C | T | G | A | C | A |  | G | A | A | C | **A** | **A** | **G** | A | **T** |
| H11 | **A** | A | T | A | A | C | T | G | A | C | A |  | G | **C** | A | C | C | **A** | T | A | a |
| H12 | **A** | **T** | T | A | **C** | C | T | G | A | C | A |  | G | A | A | C | C | C | T | A | **7** |
| H13 | **A** | A | **G** | A | A | **G** | T | G | **C** | **A** | A |  | G | A | A | C | C | C | T | A | a |
| H14 | **A** | A | T | A | A | C | T | G | A | **A** | A |  | G | A | A | C | C | C | T | A | a |
| H15 | **A** | A | T | A | A | C | T | G | A | C | A |  | G | A | **4** | C | C | C | T | A | a |
| H16 | **A** | A | T | **1** | A | C | T | G | A | C | A |  | G | A | A | C | C | **A** | **G** | A | a |
| H17 | **A** | A | T | A | A | C | **2** | G | A | C | A |  | G | A | A | C | C | **A** | T | A | a |
| H18 | **A** | A | **G** | A | A | **G** | T | G | A | **A** | A |  | G | A | A | C | C | C | T | A | a |
| H19 | **A** | A | T | A | A | C | T | G | A | C | **C** |  | G | A | **4** | **5** | C | C | T | A | a |
| H20 | **A** | A | **G** | A | A | **G** | T | G | **C** | **A** | A |  | G | A | A | C | C | C | T | **6** | a |

All haplotype sequences were specified with reference to H1; a: GCACCTTCTTGATAGAACAAGAAAATGAT

1 Indel: TTAAAACATAAAATAAAACTAAATAGATCCACTTCATTTATCACAATGAATTATATTTG; 2 Indel: AAAATAGATACAAAAAAGTCCAAAT; 3 Indel: CCCAATTGAAGGAAATG; 4 Indel: TTTCAAAGATAAGAATAT; 5 Indel: GAAGT; 6 Indel; TA; 7 inversion: ATCATTTTCTTGTTCTATCAAGAA

GGTGC.

**Table S5** Details of 69 sample locations used for ecological niche modeling analysis.

| Species | Iatitude(°N) | Longitude(°E) |
| --- | --- | --- |
| 1 | 40.25 | 75.58 |
| 2 | 39.00 | 76.27 |
| 3 | 39.32 | 77.16 |
| 4 | 39.21 | 78.03 |
| 5 | 40.29 | 78.56 |
| 6 | 41.38 | 79.09 |
| 7 | 40.07 | 79.45 |
| 8 | 41.13 | 80.21 |
| 9 | 37.20 | 80.23 |
| 10 | 41.04 | 80.29 |
| 11 | 37.01 | 81.20 |
| 12 | 40.53 | 81.29 |
| 13 | 40.41 | 81.38 |
| 14 | 40.38 | 81.51 |
| 15 | 45.27 | 82.03 |
| 16 | 45.21 | 83.04 |
| 17 | 41.22 | 83.18 |
| 18 | 40.37 | 85.52 |
| 19 | 45.96 | 85.54 |
| 20 | 48.25 | 86.07 |
| 21 | 42.12 | 86.11 |
| 22 | 40.43 | 86.18 |
| 23 | 44.29 | 86.22 |
| 24 | 42.54 | 86.24 |
| 25 | 42.31 | 86.35 |
| 26 | 42.33 | 86.53 |
| 27 | 44.18 | 87.10 |
| 28 | 44.28 | 87.25 |
| 29 | 47.43 | 87.45 |
| 30 | 47.27 | 88.10 |
| 31 | 48.43 | 88.23 |
| 32 | 41.00 | 88.30 |
| 33 | 44.39 | 89.05 |
| 34 | 44.02 | 89.52 |
| 35 | 38.46 | 94.25 |
| 36 | 38.05 | 94.53 |
| 37 | 40.52 | 95.03 |
| 38 | 40.13 | 95.11 |
| 39 | 36.37 | 95.14 |
| 40 | 40.37 | 95.23 |
| 41 | 36.39 | 95.26 |
| 42 | 38.24 | 95.36 |
| 43 | 36.44 | 96.15 |
| 44 | 40.52 | 96.18 |
| 45 | 36.40 | 96.40 |
| 46 | 36.41 | 96.45 |
| 47 | 37.01 | 96.51 |
| 48 | 37.25 | 97.23 |
| 49 | 38.24 | 97.25 |
| 50 | 37.38 | 97.34 |
| 51 | 40.21 | 97.58 |
| 52 | 40.25 | 98.18 |
| 53 | 39.37 | 99.05 |
| 54 | 40.08 | 99.13 |
| 55 | 40.37 | 99.32 |
| 56 | 39.39 | 99.44 |
| 57 | 38.00 | 100.00 |
| 58 | 39.14 | 100.40 |
| 59 | 42.30 | 101.28 |
| 60 | 42.32 | 102.03 |
| 61 | 39.03 | 103.02 |
| 62 | 39.06 | 103.12 |
| 63 | 36.05 | 103.18 |
| 64 | 39.33 | 104.07 |
| 65 | 38.50 | 105.41 |
| 66 | 37.40 | 105.70 |
| 67 | 38.03 | 106.26 |
| 68 | 38.46 | 106.26 |
| 69 | 38.01 | 109.19 |

Sample location records from the Chinese Virtual Herbarium: 85.54°E , 45.96°N; 96.4°E, 36.4°N; 100.4°E, 39.14°N; Sample location records from the Global Biodiversity Information Facility: 105.7°E, 37.4°N;
