# Supplementary material for: Phylogeography and Population Genetics Analyses Reveal Evolutionary History of the Desert Resource Plant Lycium ruthenicum (Solanaceae)
Source: Front Plant Sci. 2022 Jun 30;13:915526. doi: 10.3389/fpls.2022.915526 (PMC9280156; doi:10.3389/fpls.2022.915526)
Supplement: Supplementary file 2 [file Data_Sheet_2.zip › Data Sheet 1 (1).ZIP!/Figure pdf/Figure 1.pdf]

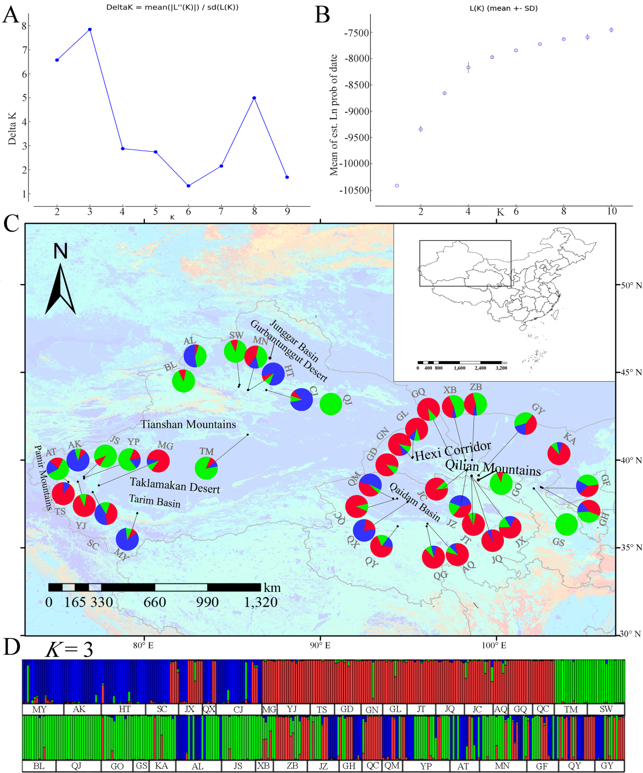
A
DeltaK = mean(|L''(K)|) / sd(L(K))
Delta K
K
B
L(K) (mean +- SD)
Mean of est. Ln prob of date
K
C
N
Junggar Basin
Gurbantunggut Desert
Tianshan Mountains
Pamir Mountains
Taklamakan Desert
Tarim Basin
Hexi Corridor
Qilian Mountains
Qaidam Basin
AL
BL
SW
MN
HT
CJ
QJ
AK
AT
JS
YP
MG
TM
TS
YJ
SC
MY
GQ
XB
ZB
GL
GN
GD
GY
KA
QM
JC
GO
GF
GH
QC
QX
JZ
JT
JX
GS
QY
QG
AQ
JQ
km
0 165 330 660 990 1,320
50° N
45° N
40° N
35° N
30° N
80° E
90° E
100° E
D
K = 3
MY AK HT SC JX QX CJ MG YJ TS GD GN GL JT JQ JC AQ GQ QC TM SW
BL QJ GO GS KA AL JS XB ZB JZ GH QC QM YP AT MN GF QY GY
