# Supplementary material for: Phylogeography and Population Genetics Analyses Reveal Evolutionary History of the Desert Resource Plant Lycium ruthenicum (Solanaceae)
Source: Front Plant Sci. 2022 Jun 30;13:915526. doi: 10.3389/fpls.2022.915526 (PMC9280156; doi:10.3389/fpls.2022.915526)
Supplement: Supplementary file 2 [file Data_Sheet_2.zip › Data Sheet 1 (1).ZIP!/Figure pdf/Figure 2.pdf]

A

B

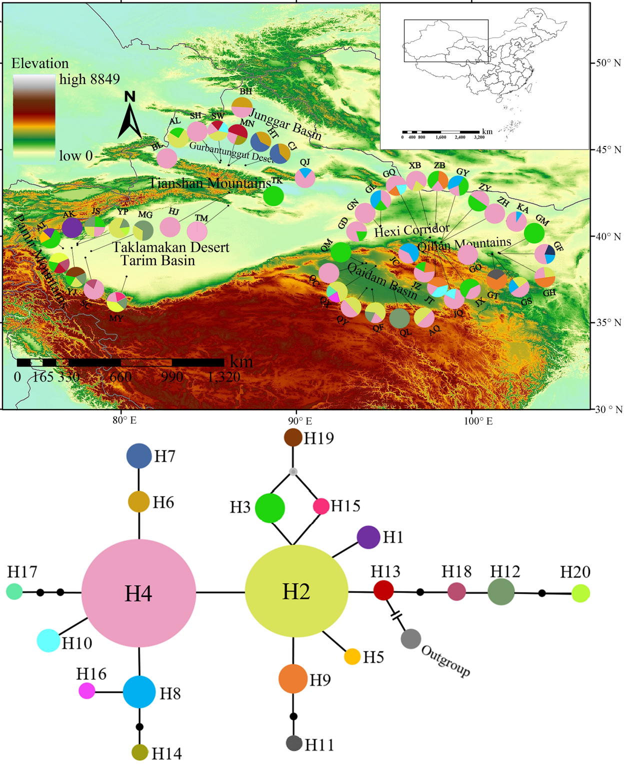

Elevation
high 8849
low 0
N
Junggar Basin
Gurbantunggut Desert
Tianshan Mountains
Pamir Mountains
Taklamakan Desert
Tarim Basin
Hexi Corridor
Qilian Mountains
Qaidam Basin
BH
AL
SH
SW
MN
HT
CJ
BL
QJ
TK
AK
JS
YP
MG
HJ
TM
AT
YJ
SC
MY
XB
ZB
GY
GQ
GL
ZY
ZH
KA
GM
GN
GD
GF
QM
JC
GO
JZ
GT
GS
GH
QC
QX
QY
QF
QL
AQ
JQ
JX
JT
km
0 165 330 660 990 1,320
50° N
45° N
40° N
35° N
30° N
80° E
90° E
100° E
0 400 800 1,600 2,400 3,200
H1
H2
H3
H4
H5
H6
H7
H8
H9
H10
H11
H12
H13
H14
H15
H16
H17
H18
H19
H20
Outgroup
