# Supplementary material for: Phylogeography and Population Genetics Analyses Reveal Evolutionary History of the Desert Resource Plant Lycium ruthenicum (Solanaceae)
Source: Front Plant Sci. 2022 Jun 30;13:915526. doi: 10.3389/fpls.2022.915526 (PMC9280156; doi:10.3389/fpls.2022.915526)
Supplement: Supplementary file 2 [file Data_Sheet_2.zip › Data Sheet 1 (1).ZIP!/Figure pdf/Figure 3.pdf]

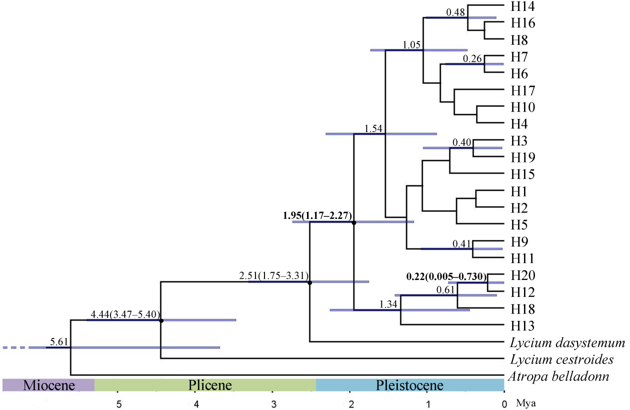

H14
0.48
H16
H8
1.05
H7
0.26
H6
H17
H10
H4
1.54
H3
0.40
H19
H15
H1
H2
1.95(1.17–2.27)
H5
H9
0.41
H11
0.22(0.005–0.730)
H20
2.51(1.75–3.31)
H12
0.61
H18
1.34
4.44(3.47–5.40)
H13
Lycium dasystemum
5.61
Lycium cestroides
Atropa belladonn
Miocene
Plicene
Pleistocene
5
4
3
2
1
0
Mya
