# Supplementary material for: Phylogeography and Population Genetics Analyses Reveal Evolutionary History of the Desert Resource Plant Lycium ruthenicum (Solanaceae)
Source: Front Plant Sci. 2022 Jun 30;13:915526. doi: 10.3389/fpls.2022.915526 (PMC9280156; doi:10.3389/fpls.2022.915526)
Supplement: Supplementary file 2 [file Data_Sheet_2.zip › Data Sheet 1 (1).ZIP!/Figure pdf/Figure 4.pdf]

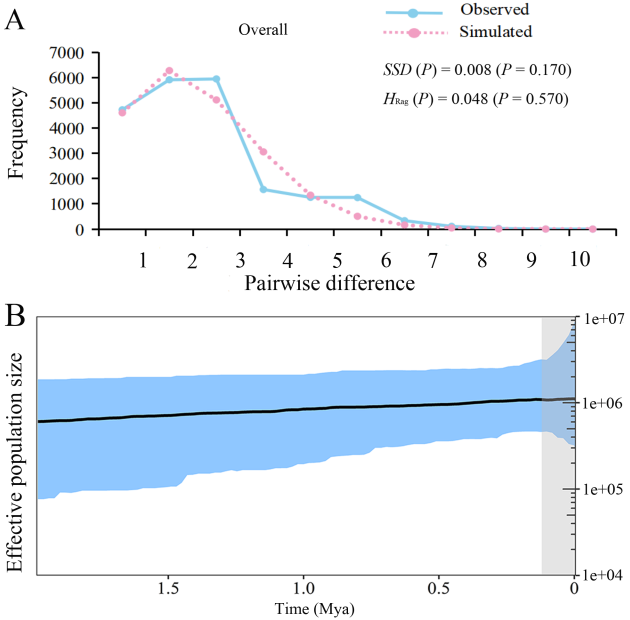

A
Overall
Observed
Simulated
SSD (P) = 0.008 (P = 0.170)
HRag (P) = 0.048 (P = 0.570)
Frequency
7000
6000
5000
4000
3000
2000
1000
0
1
2
3
4
5
6
7
8
9
10
Pairwise difference
B
Effective population size
1e+07
1e+06
1e+05
1e+04
1.5
1.0
0.5
0
Time (Mya)
