# Supplementary material for: Phylogeography and Population Genetics Analyses Reveal Evolutionary History of the Desert Resource Plant Lycium ruthenicum (Solanaceae)
Source: Front Plant Sci. 2022 Jun 30;13:915526. doi: 10.3389/fpls.2022.915526 (PMC9280156; doi:10.3389/fpls.2022.915526)
Supplement: Supplementary file 2 [file Data_Sheet_2.zip › Data Sheet 1 (1).ZIP!/Figure pdf/Figure 5.pdf]

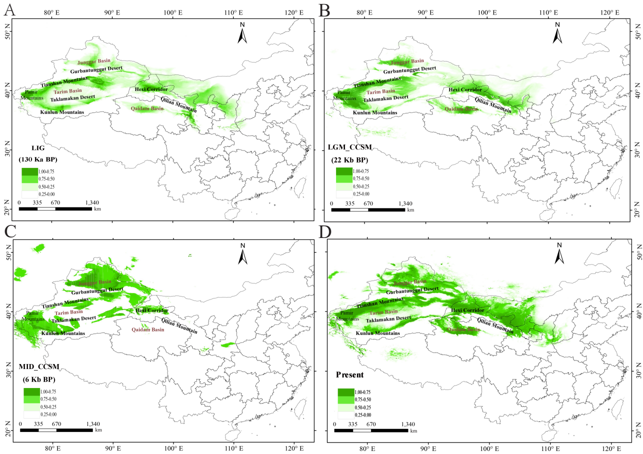

A
B
C
D
80° E
90° E
100° E
110° E
120° E
50° N
40° N
30° N
20° N
N
Junggar Basin
Gurbantunggut Desert
Tianshan Mountains
Pamir Mountains
Tarim Basin
Taklamakan Desert
Kunlun Mountains
Hexi Corridor
Qaidam Basin
Qilian Mountain
LIG
(130 Ka BP)
LGM_CCSM
(22 Kb BP)
MID_CCSM
(6 Kb BP)
Present
1.00-0.75
0.75-0.50
0.50-0.25
0.25-0.00
0
335
670
1,340
km
